# Construction of a Quantitative Acetylomic Tissue Atlas in Rice (*Oryza sativa* L.)

**DOI:** 10.3390/molecules23112843

**Published:** 2018-11-01

**Authors:** Zhiyong Li, Yifeng Wang, Babatunde Kazeem Bello, Abolore Adijat Ajadi, Xiaohong Tong, Yuxiao Chang, Jian Zhang

**Affiliations:** 1 State Key Lab of Rice Biology, China National Rice Research Institute, Hangzhou 311400, China; lzhy1418@163.com (Z.L.); wangyifeng@caas.cn (Y.W.); tunlapa2k3@yahoo.com (B.K.B.); threetriplea@yahoo.com (A.A.A.); tongxiaohong@caas.cn (X.T.); 2 Agricultural Genomes Institute at Shenzhen, Chinese Academy of Agricultural Sciences, Shenzhen 518120, China

**Keywords:** Rice (*Oryza sativa* L.), protein lysine acetylation, proteome, tissue atlas, post-translational modification

## Abstract

PKA (protein lysine acetylation) is a key post-translational modification involved in the regulation of various biological processes in rice. So far, rice acetylome data is very limited due to the highly-dynamic pattern of protein expression and PKA modification. In this study, we performed a comprehensive quantitative acetylome profile on four typical rice tissues, i.e., the callus, root, leaf, and panicle, by using a mass spectrometry (MS)-based, label-free approach. The identification of 1536 acetylsites on 1454 acetylpeptides from 890 acetylproteins represented one of the largest acetylome datasets on rice. A total of 1445 peptides on 887 proteins were differentially acetylated, and are extensively involved in protein translation, chloroplast development, and photosynthesis, flowering and pollen fertility, and root meristem activity, indicating the important roles of PKA in rice tissue development and functions. The current study provides an overall view of the acetylation events in rice tissues, as well as clues to reveal the function of PKA proteins in physiologically-relevant tissues.

## 1. Introduction

PKA (Protein lysine acetylation) refers to the substitution of an acetyl group for an active hydrogen atom on the lysine residues of a protein. Three types of proteins are required to catalyze the reversible PKA reaction. As acetylation “writers”, lysine acetyltransferases (KATs) catalyze the addition of acetyl groups from acetyl-coenzyme A (acetyl-CoA) to proteins, whereas the reversible deacetylation process is conducted by the “erasers” enzyme lysine deacetylases (KDACs). Proteins containing conserved bromodomain (BRD) or YEATS domain were found to play the roles of PKA “readers” (acetyllysine binders), as they can selectively interact with acetylated proteins [1]. Since its first discovery on histones in over 50 years ago, PKA has been implicated for the functionality of their target proteins in various cellular processes [2]. Histone acetylation has been associated with chromatin remodeling and transcription activation, because a negatively-charged acetyl group could neutralize the positive charges of lysine residues, which weakens the interaction of the histone with negatively charged DNA, and consequently leads to a more relaxed chromatin structure for transcription. Conversely, the reversible histone deacetylation usually results in a tighter interaction with DNA, leading to chromatin condensation and transcription repression [3]. Recently, knowledge regarding PKA has been extended to non-histone proteins, particularly key metabolic enzymes related to glycolysis, tricarboxylic acid (TCA) cycle in different organisms, as well as photosynthesis in plants [4,5,6,7]. The status and intensities of PKA may impose profound effects on the function of non-histone proteins by altering their enzyme activity, cellular compartment localization, protein-nucleotide/protein-protein interaction, and protein stability [3,8]. For example, the inhibition of PKA on tumor protein p53 is believed to be a cause of cervical cancer in human [9]. In Arabidopsis, Lee et al. (2015) revealed that the *P. syringae* type-III effector HopZ3, which is a YopJ type acetyltransferase, suppresses plant immune system by acetylating multiple members of the RPM1 immune complex and its triggering effectors [10].

As the first step toward understanding PKA, identification of PKA sites and dynamics is crucial. Aided by the technologies of acetylpeptides immune affinity purification and nano-HPLC/MS/MS, Kim et al. (2006) reported the first proteome-wide profiling of PKA in HeLa cells and mouse liver mitochondria. This screening identified 388 PKA sites on 195 proteins, which dramatically extended the known inventory of in vivo acetylation sites and substrates [11]. So far, the information of 111253 PKA sites on 33025 PKA proteins from various species have been deposited into the PLMD (Protein Lysine Modifications Database, http://plmd.biocuckoo.org/download.php) [12]. In comparison with the tremendous progress achieved in human, mouse, fungi, and bacterium, PKA identification in plants is lagging behind. Until 2011, Finkemeier et al. reported the first plant acetylomic analysis in the dicot model species Arabidopsis. They revealed the extensive involvement of PKA in regulating the activity of central metabolic enzymes such as Rubisco, phosphoglycerate kinase, glyceraldehyde 3-phosphate dehydrogenase, and malate dehydrogenase [13]. Nevertheless, only two reports are presently available on Arabidopsis, which profiled a total of 398 PKA sites on 251 proteins from suspension cells and young seedlings [13,14]. Similar works have also been done on grape fruits, pea seedlings, soybean developing seeds, wheat leaf, strawberry leaf, grass leaf, potato tuber, and spruce somatic embryo, but only yielded the identification of less than 7000 PKA sites in total [6,15,16,17,18,19,20,21]. 

Rice (*Oryza sativa* L.) is one of the most important food crops, as it serves as a staple food for over half of the global population. On the other hand, rice is also a model species for biological research due to its relatively small genome size, released genome sequence, ample genetic resources, as well as the co-linearity with other grasses [22]. PKA has been recognized as a key mechanism in regulating photosynthesis and metabolism in plants. For example, acetylation of the Lhcb1 and Lhcb2 proteins appear to be involved in determining the LHC attachment to PSII complexes. Higher PKA level on Lhcbs impaired their binding ablity to PSII to form the PSII-LHCII supercomplexes [23]. This also intrigued researchers to explore PKAs in rice. So far, at least 8 independent cases have been reported regarding the profiling of PKA sites, peptides, and proteins in rice seedlings, reproductive organs, and leaves under oxidative stress, which yielded the identification of over 8599 PKA sites and 4990 proteins [7,24,25,26,27,28,29,30]. Despite efforts being made on rice, the previous studies only explored the tip of the iceberg of rice acetylome, given that rice genome contains over 56,000 protein coding genes [31]. Due to the spatial- and temporal-pattern of protein expression, investigating PKA in various tissues has been considered as an effective way to expand the coverage of rice acetylome. In this study, we performed a quantitative, MS-based identification of PKA proteins in four rice tissues, i.e., the callus, root, leaf, and panicle, and obtained 890 PKA proteins covering 1536 sites, which allowed us to construct a tissue atlas of rice acetylome. The results obtained from this work aimed to provide an overall view of the acetylation events in rice tissues, as well as clues to reveal the function of PKA proteins in physiologically-relevant tissues.

## 2. Results

### 2.1. Profiling the Acetylsites and Acetylproteins on Various Rice Tissues

**Figure 1:**
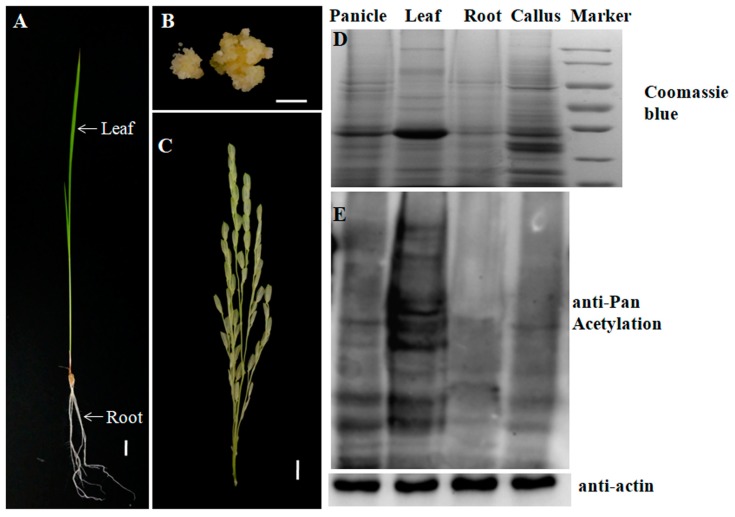
Tissue morphologies of different rice tissues tested in this study. (**A**) leaf and root; (**B**) callus; (**C**) mature panicle. Scale bar = 1 cm; (**D**) Total proteins of different tissues were resolved by SDS-PAGE and stained by Coomassie brilliant blue (CBB) and (**E**) Western blot analysis of the acetylation dynamics in different rice tissues by using anti-acetyl lysine antibodies. Equal amount of proteins (20 μg) were used. Anti-actin was used as an internal control for normalization [32].

**Figure 2:**
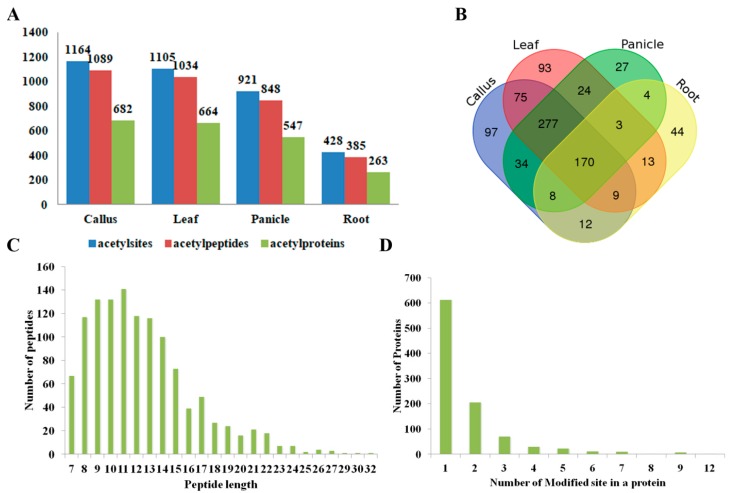
(**A**) The counts of acetylsites, acetylpeptides and acetylproteins in the callus, leaf, panicle, and root, respectively; (**B**) Venn diagram showing the overlap of our identified acetylproteins in the callus, leaf, panicle, and root, respectively; (**C**) Numbers of each identified peptide length; (**D**) Numbers of each identified modified site in a protein.

PKA is a highly transient and reversible post-translational modification. To achieve an overview of PKAs in rice, we conducted a comprehensive, quantitative acetylomic profiling in 4 tissues of Nipponbare (*Oryza sativa* L. *ssp japonica*), i.e., callus, leaves, panicles, and roots (Figure 1A–C), by employing a label-free, MS-based approach. Prior to the MS identification, we performed western blot analysis on the tissue proteins using a pan anti-acetylation antibody. The protein acetylation levels and patterns varied divergently from tissues, and the majority of the acetylated proteins are non-histone proteins with a molecular weight over 25 KD (Figure 1D,E). MS identification yielded the data of 1164, 1105, 921, and 428 acetylsites on 1089, 1034, 848, and 385 acetylpeptides, which represented 682, 664, 547, and 263 acetylproteins from the callus, leaves, panicles and roots, respectively (Figure 2A, Table 1 and Table S1). One hundred and seventy acetylproteins were commonly profiled in all the four tissues, while 97, 93, 27, and 44 acetylproteins were specifically detected in the callus, leaves, panicles, and roots, respectively (Figure 2B). By integrating the data from four tissues and removing redundancies, a total of 1454 acetylpeptides from 890 acetylproteins, covering 1536 acetylsites, were obtained. The mass errors for those acetylpeptides were near 0, with the majority less than 5 ppm, implying a high level of accuracy of the data (Figure S1). The length of most of the identified acetylpeptides ranged from 7–22 amino acids with a few longer exceptions that even reached up to 32 amino acids (AAs) (Figure 2C). Over 60% of the acetylpeptides carried only 1 acetylsite, around 20% of the acetylpeptides covered 2 or 3 acetylsites, while the remaining 10% acetylpeptides had more than 3 acetylsites (Figure 2D).

### 2.2. Motif Analysis of the Acetylsite Flanking Sequences

**Figure 3:**
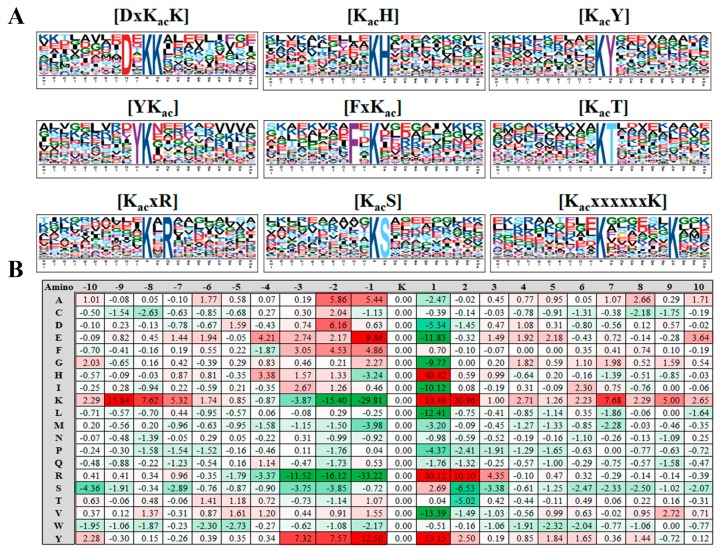
(**A**) Acetylation sequence motifs and conservation of acetylation sites in identified callus, leaf, panicle acetylproteins; and (**B**) Heat map of the amino acid composition of the acetylation sites, showing the frequency of different amino acids surrounding the acetylated lysine (K).

Employing the motif-X algorithm (http://motif-x.med.harvard.edu/) [33], we searched the potential conserved motifs in the context of 21 AA-long sequences centered by the acetylsites. Nine of the most enriched motifs are depicted in Figure 3A (Fold increase > 2.5, P < 0.01). Positively-charged residues like K and H are greatly favored by PKA, as we found the top three enriched consensus motifs were [DxK_ac_K,K_ac_H] and [K_ac_K]. There were 132, 118, and 73 acetylsites located in [K_ac_xR,K_ac_S] and [K_ac_T] motifs respectively. Meanwhile, motifs [YK_ac_] [FxK_ac_] [KxxxxxxK_ac_] also showed over 2.5 fold enrichment when compared with the rice proteome background. In addition to the conserved motifs, we calculated the frequency of each AA type in the positions flanking the acetylsites. As revealed in Figure 3B, the distribution of AAs displayed strong bias in certain positions. For example, there was significantly higher chance of finding a H, K, R, or Y in the +1 position to acetylsites, while residues like M, I, G, and E were barely detected in the same position.

### 2.3. Functional Features of Rice Acetylproteins

**Figure 4:**
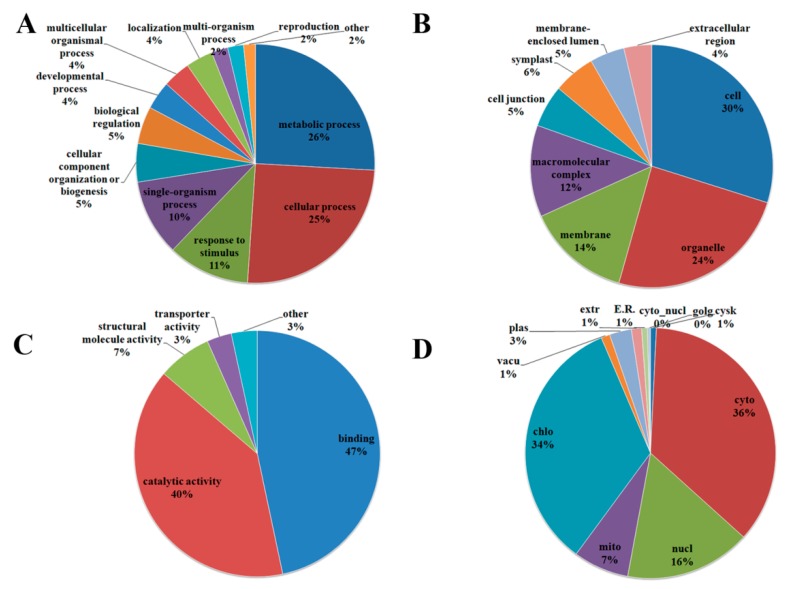
GO analysis of differentially-acetylated proteins in terms of: biological process (**A**); molecular function (**B**); cellular component (**C**); subcellular location (**D**); and acetylation intensity, respectively. The abbreviations in (**D**) represent the following. E.R.: endoplasmic reticulum; extr: extracellular matrix; cyto: cytoplasm; mito: mitochondrial; chlo: chloroplast; vacu: vacuole; cysk: cytoplasmic skeleton; cyto nucl: cytoplasm nuclear.

GO functional classification of all the acetylproteins were investigated in the terms of “biological process”, “cellular component”, and “molecular function”. For the “biological process” category, the largest two types of acetylprotein was associated with “metabolic process” and “cellular process”, which accounted for over half of the identified acetylproteins, suggesting a predominant role of PKA in metabolism. The remaining acetylproteins were related to processes such as “response to stimulus”, “single-organism process”, and “cellular component organization or biogenesis” (Figure 4A). Within the category of “cellular component”, 30%, 24%, and 14% of the acetylproteins were annotated as “cell”, “organelle”, and “membrane”, respectively. Meanwhile, we found that a small portion of the proteins were relevant to “macromolecular complex”, “cell junction”, and so on (Figure 4B). Consistent with the highly-presented “metabolic process”, the “molecular function” of the majority of the acetylproteins was on “binding” (47%) and “catalytic activity” (40%), while the remaining were likely to be involved in “structural molecule activity” and “transporter activity” (Figure 4C).

Eukaryotic cells are comprised of several membrane-bound subcellular compartments, such as the nucleus, cytoplasm, and mitochondria, where functional proteins play distinct roles [34]. The prediction of the subcellular localization helps to understand the function of the acetylproteins. We revealed that most of the acetylproteins were located to the cytosol (36%) and chloroplast (34%) (Figure 4D). Nuclear proteins such as histones and transcription factors are regarded as potential regulators of gene transcriptions. Sixteen percent of our identified acetylproteins were localized to nuclear, indicating that PKA is extensively involved in gene regulation. Some of the acetylproteins were predicted to be in mitochondria (7%), plasma membrane (3%), cytoskeleton (2%), and vacuole (1%) (Figure 4D).

**Figure 5:**
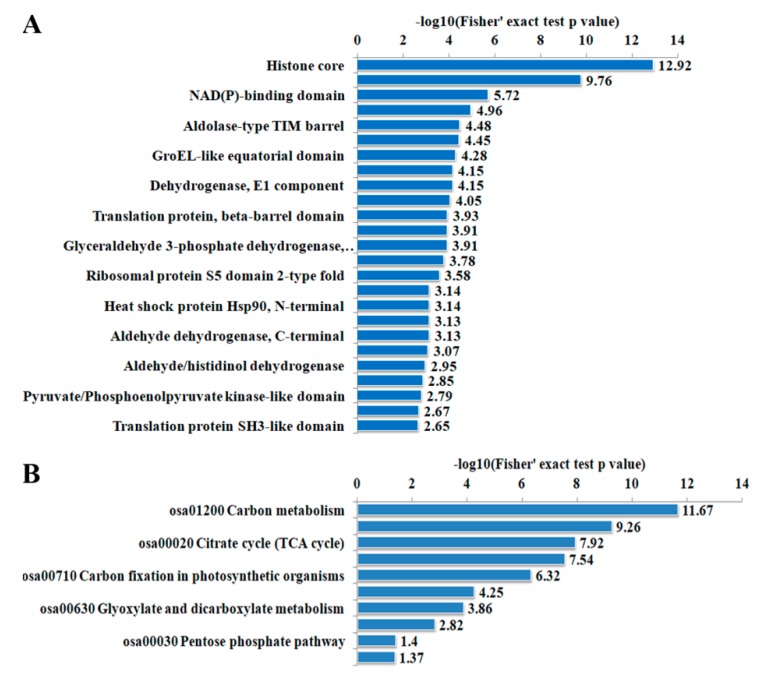
Protein domain enrichment analysis (**A**) and KEGG pathway enrichment analysis (**B**) proteins identified acetylproteins in this study.

Functional domains on a protein usually indicated its function in biological processes. In this study, we performed a PFAM functional domain enrichment assay to address the functional domain features of rice acetylproteins (Figure 5A). As a result, the most enriched proteins are core histones and histone-fold proteins, indicating PKA occurrs extensively on histones. Other enriched functional domains include NAD(P)-binding domain, single hybrid motif, aldolase-type TIM barrel, enhancer of polycomb-like and groEL-like equatorial domains, which are extensively involved in metabolism, and transcriptional and translational regulations.

We further conducted KEGG pathway enrichment analysis of the acetylproteins. The top 5 over-presented pathways are carbon metabolism, glycolysis, TCA cycle, biosynthesis of amino acides, and carbon fixation in photosynthetic organisms, which is in line with the functional domain analysis result that PKA occurs extensively on metabolism-related proteins (Figure 5B).

### 2.4. Differentially Acetylated (DA) Peptides among Rice Tissues

**Figure 6:**
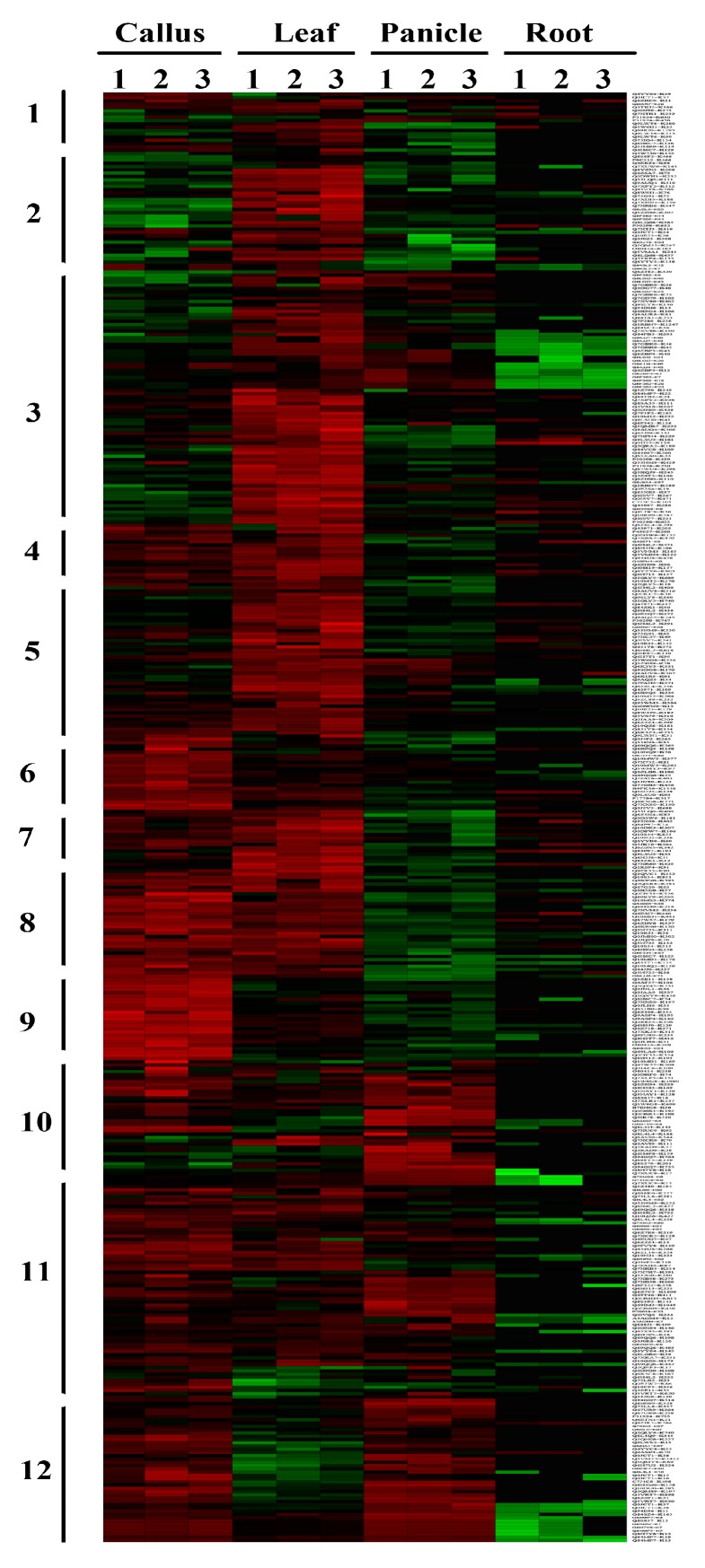
Hierarchical clustering analysis of the DA proteins in the callus, leaf, panicle, and root; Color bar at the bottom represents the log 2 acetylation site quantitation values. Green, black, and red indicate the low, medium, and high acetylation intensity, respectively.

Employing a label-free approach, we quantified the acetylation intensity in each peptide, and identified 1445 DA peptides on 887 proteins (Table S2). These included 125, 113, 35, and 53 peptides which were specifically acetylated in the callus, leaf, panicle, and root, respectively. In addition, we found 27 peptides were commonly acetylated in all the four tested tissues, with no significant variations in the acetylation intensity level, indicating the potential use as an internal control for PKA quantification experiments among rice tissues. There were also 82 acetylpeptides that were constitutively acetylated with significantly different intensities in the four tissues. The acetylation intensities of each DA proteins in various tissues were further visualized in a heatmap (Figure 6). The 1445 DA peptides were primarily divided into 12 clusters; each represented a divergent acetylation pattern from the others. For example, DA peptides in cluster 5 are predominantly acetylated in leaf, while cluster 9 contains peptides that are predominantly acetylated in the callus. In cluster 8, the peptides are highly acetylated in both the callus and leaf, suggesting the PKA on these peptides may regulate pathways that are common to the two tissues.

### 2.5. Protein-Protein Interaction (PPI) Analysis of Acetylproteins

**Figure 7:**
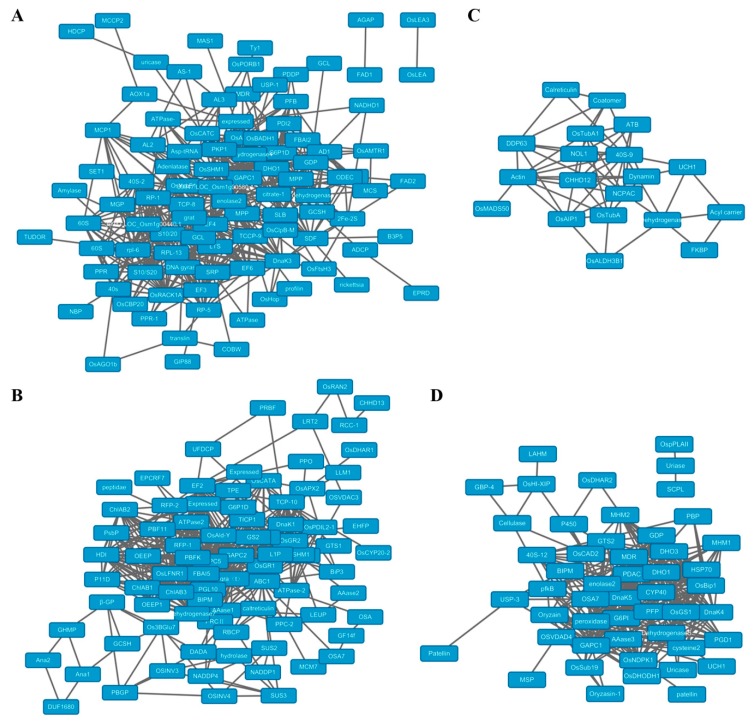
Protein–protein interaction (PPI) network of DA proteins identified in this study. (**A**) callus; (**B**) leaf; (**C**) mature panicle, and (**D**) root.

Using STRING (Search Tool for the Retrieval of Interacting Genes/Proteins version 10.0; http://string-db.org/) and Cytoscape software (Cytoscape 3.7.0, Bethesda, Rockville, MD, USA), we constructed protein-protein interaction (PPI) networks of the specifically-acetylated proteins in the four tissues for a better understanding of the possible relationships among them. The callus acetylproteins form a profound PPI network containing 98 nodes (proteins) and 532 edges (interaction-ships) (Figure 7A; Table S3). In this network, we identified 10 ribosomal proteins, including 40S ribosomal protein S24, S9-2, 60S ribosomal protein L27, L36, as well as some ribosome recycling factors, which may form a ribosomal complex and take charge of the protein translation in callus. For the leaf acetylproteins, we acquired a network with 85 nodes and 465 edges (Figure 7B). Photosynthesis-related proteins were involved in this network. For examples, three chlorophyll A–B binding proteins, which serve as light receptors in the light-harvesting complex (LHC), may interact with each other. The network also contains carbohydrates assimilation proteins such as sucrose synthases and exo-beta-glucanase. In addition, we revealed an interaction module of gra(t)-OsAld-OsPORB, in which all members are functionally related to chloroplast development. In the network of panicle-acetylated proteins, an actin protein was associated with rice flowering date regulator OsMADS50 and pollen fertility regulator OsAIP1, suggesting that PKA on these proteins plays important roles in flower development (Figure 7C). The network identified from root-acetylated proteins is majorly implicated in pentose metabolism, as a couple of 6-phosphogluconate dehydrogenases, dihydroxy-acid dehydratases, and glutamate dehydrogenases were involved in it (Figure 7D).

## 3. Discussion

### 3.1. PKA Profiling in Rice

Given the essential regulatory roles of PKA in plant growth and development, acetylomic profiling has become a hot topic in plant research. To date, at least 8 PKA identification cases have been reported in rice, as well as many in other plant species (Table 2). Due to its easy availability, vegetative tissues like leaf or whole seedlings have been repeatedly used for acetylomic identification. Xiong et al. (2016) profiled a total of 716 rice seedling PKA proteins, which were implicated in glyoxylate and dicarboxylate metabolism, carbon metabolism, and photosynthesis pathways [7]. Later, Xue et al. (2017) further identified 866 acetylproteins by using the same seedling tissue. However, only 21.3% of the acetylproteins were overlapped with the previous study [29]. The authors deduced that the difference in identification is due to the physiological status or growth stages of the samples used. Zhou et al. (2018) also mapped 1024 acetylproteins from rice leaves under oxidative stress [30]. In rice germinating embryo, 699 PKA sites on 389 proteins were found to be acetylated, including 144 sites that were simultaneously modified by succinylation [26]. In an effort to discover the PKA in rice anthers in the stage of meiosis, Li et al. (2018) identified a total of 1354 PKA sites in 676 proteins, which are mostly related to chromatin silencing, protein folding, and fatty acid biosynthetic processes [27]. There were also 44 and 692 lysine acetylated proteins that were identified from suspension cells and grain-filling stage seeds [24,28]. With a special focus on the seed development, Wang et al. (2017) carried out a quantitative acetylproteomic study on pistil and early developing seeds. A total of 972 acetylproteins harboring 1817 acetylsites were identified, and 268 acetylproteins were differentially acetylated in the three developing stages, providing novel insight into PKA in rice seed development [25]. Nevertheless, rice acetylome is extremely complicated, largely due to the dynamic expression pattern of proteins in various tissues and physiological conditions. To gain a more complete coverage of the rice acetylome, the current study quantitatively profiled a total of 1454 acetylpeptides from 890 acetylproteins, covering 1536 acetylsites from callus, leaf, panicle, and root, which represented 4 major tissue types in rice. In comparison with the previous data, around 85% of our identified acetylprotiens were overlapped with other studies. A few examples are MADS50 (Q9XJ60), SUS1 (P31924), and USP (Q5Z8Y4). Meanwhile, we also found that 139 acetylproteins are novel, which were majorly identified from previously untested tissues such as the callus and panicle (Table S1). Our dataset greatly expanded the rice acetylome inventory, considering that callus and panicle are two unexplored tissues in terms of acetylome profiling. In contrast to previous studies that used the whole seedling for profiling, our dataset separated the tissues into leaf and root, thus presenting more detailed information of the spatial-distribution of the acetylproteins. Moreover, the quantified protein acetylation intensities also provided cues for functional characterization of the acetylproteins in the development of the corresponding tissues.

### 3.2. Acetylproteins in Rice Callus

Rice callus is a mass of unorganized parenchyma cells which is capable of being regenerated in an individual plant. Callus cells have robust metabolisms and protein expressions to maintain their capability for differentiation. Ribosome is a complex molecular machine known as the translational apparatus for protein synthesis. Ribosome consists of two major components: small ribosomal subunits for RNA recognition, and large ribosomal subunits for the synthesis of polypeptide chain using amino acid substrates. Early in the 1970s, PKA was found to occur on rabbit ribosomal proteins, and was implicated in the formation of the initiation complex during translation [37]. In yeast, protein N-terminal acetylation of ribosomal proteins by *N*-acetyltransferase is necessary to maintain protein synthesis [38]. It seems that PKA on ribosomal proteins might be a conserved mechanism, as we found that 41 ribosomal proteins were acetylated in rice callus. Interestingly, twelve of these proteins were predominantly acetylated in callus but not in the other tested tissues, including 40S ribosomal proteins S4 (Q0E4Q0), S9 (Q2R1J8), S20 (Q10P27), and S24 (Q0DBK8), as well as 60S ribosomal proteins L2 (P92812), L21 (Q10RZ3), L27 (Q7XC31), and L36 (Q6L510), suggesting their essential roles in protein expression regulation in callus (Table S1). In addition to the ribosomal proteins, OASA1 (Q94GF1), which is a key enzyme for the synthesis of indole-3-acetic acid, and OsGH3.8 (Q0D4Z6), which is involved in the auxin homeostasis, were both more acetylated in callus than in other tissues [39,40]. Thus, the auxin level may subject to PKA regulation in callus. 

### 3.3. PKA on Chloroplast Development and Photosynthesis Proteins in Leaf

Photosynthesis majorly occurs in the chloroplast of leaf, where the photosynthetic pigment chlorophyll captures and converts the light energy into the energy storage molecules ATP and NAPDH, which could be further converted into chemical energy in the form of carbohydrates, via the famous Calvin cycle. A number of key regulators of chloroplast development have been identified as acetylproteins in the current study. Fd-GOGAT1 (Q69RJ0) is a ferredoxin-dependent glutamate synthase which is known as a key enzyme in the process of inorganic nitrogen assimilation. Mutants of Fd-GOGAT1 exhibited defective chlorophyll synthesis and low photosynthesis rates, and finally led to early leaf senescence [41]. PORB (Q8W3D9) is defined as a NADPH:protochlorophyllide oxidoreductase catalyzing the photoreduction of protochlorophyllide to chlorophyllide in synthesis. Under high light conditions, PORB is essential for maintaining light-dependent chlorophyll synthesis, while disruption of the gene could turn the new leaves yellow and cause lesions [42]. WSL12 (Q0IMS5) is a chloroplast-localized, nucleoside diphosphate kinase knock-down of WLS12, which results in abnormal chloroplast and white stripes on leaves [43]. Interestingly, these three proteins were all predominantly acetylated in leaves, implying that PKA is a key switch for chloroplast development in rice.

In terms of photosynthesis, we found that numerous key enzymes were predominantly acetylated in leaf. For examples, glycine decarboxylase complex (GDC) recovers carbon following the oxygenation reaction of ribulose-1,5-bisphosphate carboxylase/oxygenase in the photorespiratory C2 cycle of C3 species [44]. LFNR (Leaf-type ferredoxin-NADP (+) oxidoreductase), an essential chloroplast enzyme, functions in the last step of photosynthetic linear electron transfer [45]. The current study revealed that rice GDCH (A3C6G9) was acetylated at the 156th lysine site, while LFNR1 (Q0DF89) harbored acetylation modifications on 79th and 188th lysine, and their acetylation intensities in leaf were significantly higher than those in other tissues. In addition, Chlorophyll A-B binding proteins (Q5ZA98, Q6Z411 and Q53N82), which are responsible for the capturing and delivering of excitation energy to photosystems [46], as well as sucrose synthases participating in starch metabolism (P31924, P30298 and Q43009), also showed similar acetylated patterns in leaf, suggesting the extensive regulatory roles of PKA on photosynthesis in leaf.

### 3.4. PKA May Regulate Flowering and Pollen Fertility in Rice Panicle

Initiation of panicle represents the transition of rice from vegetative growth to reproductive growth. Transcription factor OsMADS50 is a known key regulator in rice flowering [47]. Suppression of OsMADS50 significantly delayed rice flowering and increased the number of elongated internodes. Meanwhile, ectopical expression of this gene could even induce the initiation of inflorescence on the callus. OsMADS50 may be functionally antagonistic with another flowering repressor, OsMADS56, and operates upstream of the rice florigen gene *Hd3a* to control rice flowering [47,48]. We found an acetylation modification of OsMADS50 on the 107th lysine site, which is located in the conserved DNA binding domain MADS box (17–135 in protein sequence). It would be interesting to explore the effects of acetylation on the binding ability of OsMADS50 to its target site in future study.

Programmed cell death (PCD) of tapetum cells in anthers is critical for the proper development of male gametophytes in rice. Apoptosis inhibitor 5 (API5, Q5JK84) encoding a putative antiapoptosis protein promotes the degeneration of the tapetum by interacting with two DEAD-box ATP-dependent RNA helicases, AIP1 and AIP2. *api5* mutants were fully sterile due to the inhibited tapetal PCD process, suggesting their key roles in the development of male gametophytes [49]. Our MS identification results showed that lysine 59, 144, and 161 were acetylated on API5. However, the three acetylsites displayed various acetylation patterns in tissues. The acetylation intensities on lysine 59 and 144 are significantly higher than in other tissues, whereas lysine 161 has the highest acetylation level in leaves, although it is also moderately acetylated in the panicles. Therefore, the indication is that PKA on lysine 59 and 144, other than on lysine 161, is more likely to be involved in pollen development.

### 3.5. Acetylation on Root Proteins

In vascular plants, the root is an important, specified organ, providing mechanic anchoring strength as well as controlling the water and nutrient uptake from soil. Root meristem activity is the key to root growth and architecture. An enzyme 3-hydroxyacyl-CoA dehydrogenase named as AIM1 (Q8W1L6) was found to be a vital regulator of root meristem activity in rice. aim1 mutant displayed shortened roots with reduced salicylic acid and ROS level. It is proposed that AIM1 mediates the salicylic acid biosynthesis to promote ROS accumulation, thereby maintaining the root meristem in an active status [50]. It is interesting to note that PKA only occurred on lysines 361 and 663 of AIM1 in the callus, leaf, and panicle, but AIM1 acetylation was not found in roots, where it is expected to be functional. The observation suggested that PKA on AIM1 may have negative effects on its function.

## 4. Materials and Methods

### 4.1. Collection and Preparation of Plant Materials

The Nipponbare (*Oryza sativa* L. *ssp jing*) plants used in this study were hydroponically-cultured or grown in the field of the China National Rice Research Institute (CNRRI). Roots were harvested from 14 days after germination of the hydroponic seedlings, leaves and panicles were collected from plants in the field at 3–5 days before heading, when the husk of spikelets were in pale color, and calluses were induced as described [51]. Three biological replicates of the callus, root, leaf, and panicle were harvested and immediately stored in liquid nitrogen before use.

### 4.2. Protein Extraction

Samples was ground into fine powders in liquid nitrogen, transferred to a 5 mL centrifuge tube containing lysis buffer (8 M urea, 150 mM Tris-HCl pH 8.0, 1 mM phenylmethylsulfonyl fluoride and 1× phosphoprotein protease inhibitor complex), and shaken for 30 min on ice. The extracted proteins were sheared by sonication (Biosafer 650-92 model, Scientz, Ningbo, China) on ice before centrifugation; then, the protein in the supernatant was precipitated in cold acetone for 30 min. The protein pellets were washed in 75% ethanol, and finally dissolved in PBS (Phosphate Buffer Saline) buffer (137 mM NaCl, 2.7 mM KCl, 10 mM Na_2_HPO_4_, 2 mM KH_2_PO_4_). Qubit 2.0 fluorometer (Invitrogen, Carlsbad, CA, USA) was used for the quantification of the extracted total proteins.

### 4.3. Western Blotting

In brief, around 20 μg of each extracted total proteins was separated by 10% SDS–polyacrylamide gels, and subsequently transferred to a polyvinylidine fluoride fluoropolymer (PVDF) membrane (0.45 μm, Millipore, Darmstadt, Germany) using a Trans-Blot Turbo transfer system (Bio-Rad, Hercules, CA, USA). The TBST (10 mM Tris-HCl, 150 mM NaCl, and 0.05% Tween 20, pH 8.0) containing 5% BSA was used to block the transferred membrane for overnight at 4 °C. Acetyl lysine primary antibodies (1:1000 dilution in TBST) (ImmuneChem, Burnaby, BC, Canada) and secondary antibodies HRP (horseradish peroxidase–conjugated) (1:1000 dilution in TBST) (Beyotime Company, Shanghai, China) were used to detect the target protein bands, and lastly, visualized using the enhanced chemiluminescence (Pierce, Waltham, WA, USA).

### 4.4. Protein Digestion and Acetylpeptide Enrichment

Protein was treated by a sequential of 10 mM DTT (dl-Dithiothreitol) reduction for 1 h at 37 °C, 20 mM IAA (indole-3-acetic acid) alkylation for 45 min at room temperature in darkness, 100 mM NH_4_CO_3_ dilution and trypsin at pH 8.0 (enzyme:protein = 1:50) digestion for overnight. For acetylpeptide enrichment, the digested peptides were dissolved with NETN buffer (100 mM NaCl, 1 mM EDTA, 50 mM Tris-HCl, 0.5% NP-40, pH 8.0). The tryptic peptides were incubated with pre-washed antibody beads (PTM Biolabs, Hangzhou, China) by gently shaking at 4 °C overnight to enrich PKA peptides. Afterwards, the beads were gently washed four times with NETN buffer, and twice with ddH_2_O. Finally, the bound acetylproteins were eluted with 0.1% TFA, subsequently vacuum dried using a SpeedVac (Thermo, Waltham, MA, USA), and cleaned with C18 ZipTips (Millipore, Darmstadt, Germany) before LC–MS/MS analysis.

### 4.5. LC–MS/MS Analyses

The procedures were done as described by Wang et al. [25]. In brief, peptides were dissolved in 0.1% formic acid, and directly loaded onto a reversed-phase pre-column (Thermo, Waltham, MA, USA.). Peptide separation was performed using a reversed-phase analytical column (Thermo, Waltham, MA, USA.). The gradient for MS analysis was described as follows: starting from 6 to 22% solvent B (0.1% FA in 98% ACN) for 24 min, then 22 to 40% for 8 min, 80% over 2 min, and lastly at 80% for 5 min at a constant flow rate of 300 μL/min on an EASY-nLC 1000 UPLC system coupled with a Q ExactiveTM mass spectrometer (Thermo, Waltham, MA, USA) over a mass range of 350–1800 *m*/*z* with a resolution of 7000. Subsequently, raw data were processed for acetylpeptide identification and acetylsite quantification with MaxQuant search engine against the uniprot_Oryza sativa database. In the MaxQuant searches for carbamidomethylation on Cys, oxidation on Met, Acetylation on Lys protein-N term was set as variable modification, and 4 missed cleavages on trypsin/P were allowed. Peptide mass error was set at 10 ppm for precursor ions and 0.02 Da for fragment ions; the minimum peptide length was set at 7. The false discovery rates (FDR) were set to <1%, and the minimum score for modified peptides was set >40 for peptide identification for all searches.

## Figures and Tables

**Table 1:** Some of the selected examples of the identified acetylproteins.

Identification				Intensity on Average			
Protein Accession	Protein Names	Modified Sequence	Protein Annotation	Cal	Lea	Pan	Root
Q9XJ60	MADS50	_LEALETYK(ac)R_	MADS-box transcription factor 50			1	
Q10N21	APX1	_PLVEK(ac)YAADEK_	l-ascorbate peroxidase 1, cytosolic	1.9	1.93	1.32	0.5
Q8W3D9	PORB	_ELLADLTSSDYPSK(ac)R_	Protochlorophyllide reductase B		1		
Q69RJ0	Fd-GOGAT1	_TDILK(ac)AK_	Ferredoxin-dependent glutamate synthase	0.38	1.83		
P30298	SUS2	_IYEK(ac)YTWK_	Sucrose synthase 2	0.78	3.83	0.82	1.41
Q0IMS5	WSL12	_NVVHGSDSPDNGK(ac)R_	Nucleoside diphosphate kinase	0.82	2.27		
Q2RAK2	OsPK1	_VFNQDLYFK(ac)R_	Pyruvate kinase	2.41		0.6	1.46
Q5JK84	API5	_DFLLK(ac)PELLR_	DEAD-box ATP-dependent RNA helicase			1.57	
P31924	SUS1	_IEEK(ac)YTWK_	Sucrose synthase 1	1.48	0.91	2.1	1.09
Q93X08	UGP	_TNPSNPSIELGPEFK(ac)K_	UDP-glucose pyrophosphorylase protein	1.38	2.21	0.79	1.56
A3C6G9	GDCSH	_YTK(ac)HCEEEDAH_	Glycine cleavage system H protein		0.97	0.03	
Q53LQ0	PDIL1-1	_SPEDATNLIDDK(ac)K_	Protein disulfide isomerase-like 1-1	0.55	2.84		1.09
Q0D9D0	SBE1	_CLIEK(ac)HEGGLEEFSK_	Glycoside Hydrolase	1.29	0.78	0.83	
Q43009	SUS3	_AEK(ac)HLAGITADTPYSEFHHR_	Sucrose synthase 3		2.08		
Q5Z8Y4	USP	_THGAISEFVNPK(ac)YTDSTK_	UDP-sugar pyrophosphorylase	1.79	3.59	1.07	
Q69V57	OsAld-Y	_(ac)SAFVGK(ac)YADELIK_	Fructose-bisphosphate aldolase	2.1	1.32	0.34	
Q8W1L6	AIM1	_YTK(ac)HCEEEDAH_	Peroxisomal fatty acid beta-oxidation	5.16	2.57	1.24	
Q7GD79	RAN2	_LTYK(ac)NVPTWHR_	GTP-binding nuclear protein Ran-2	0.96	1.8	1.16	

**Table 2:** Summary of the reported acetylome identification cases in rice and other plant species.

Species	Tissue	Treatment	Acetylsites	Acetylproteins	Reference
*Oryza sativa*	Callus, root, leaf and panicle		1536	890	This study
*Oryza sativa*	Seed		1003	692	[28]
*Oryza sativa*	Pistil and developing seeds		1817	972	[25]
*Oryza sativa*	Suspension cells		60	44	[24]
*Oryza sativa*	Young seedling		1337	716	[7]
*Oryza sativa*	Leaf	Oxidative stress	1669	1024	[30]
*Oryza sativa*	Seedling		1353	866	[29]
*Oryza sativa*	Anther		1354	676	[27]
*Oryza sativa*	Seed	Imbibition	699	389	[26]
*Zea may*	Seedling	Fungus	2791	912	[35]
*Triticum aestivum*	Leaf		416	277	[6]
*Glycine max*	Seed		400	245	[17]
*Arabidopsis*	Mitochondria of leaf		243	120	[14]
*Arabidopsis*	Chloroplast of leaf		91	74	[13]
*Arabidopsis*	Leaf	Deacetylase inhibitor	2057	1022	[36]
*Arabidopsis*	Root, leaf, shoot, flower and silique		64	57	[23]

## Supplementary Materials

The supplementary materials are available online.
