# Supplementary figures and images for: Construction of a Quantitative Acetylomic Tissue Atlas in Rice (Oryza sativa L.)
Source: Molecules. 2018 Nov 1;23(11):2843. doi: 10.3390/molecules23112843 (PMC6278296; doi:10.3390/molecules23112843)

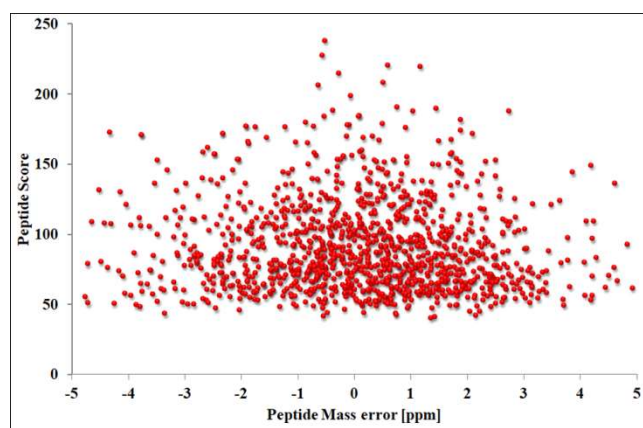

**Figure S1.** The mass errors of the DA proteins identified in this study.

Supplement: Supplementary file 1 [file molecules-23-02843-s001.zip › molecules-377239-proofreading-SM/Supplementary files/molecules-377239-proofreading-SM.pdf]

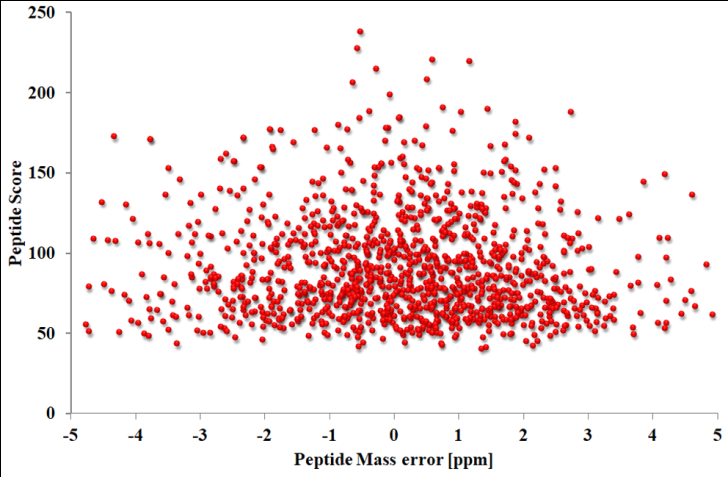


**Figure S1**. The mass errors of the DA proteins identified in this study.

Supplement: Supplementary file 1 [file molecules-23-02843-s001.zip › Supplementary files/Figure S1.docx]
